# Enhancing for Bagasse Enzymolysis via Intercrystalline Swelling of Cellulose Combined with Hydrolysis and Oxidation

**DOI:** 10.3390/polym14173587

**Published:** 2022-08-30

**Authors:** Feitian Bai, Tengteng Dong, Zheng Zhou, Wei Chen, Chenchen Cai, Xusheng Li

**Affiliations:** 1 School of Light Industrial and Food Engineering, Guangxi University, Nanning 530004, China; 2 Creating New Greatness Advanced Material Co., Ltd., Changsha 410600, China

**Keywords:** cellulose, swelling, bagasse, enzymolysis, cellulase

## Abstract

To overcome the biological barriers formed by the lignin–carbohydrate complex for releasing fermentable sugars from cellulose by enzymolysis is both imperative and challenging. In this study, a strategy of intergranular swelling of cellulose combined with hydrolysis and oxidation was demonstrated. Pretreatment of the bagasse was evaluated by one bath treatment with phosphoric acid and hydrogen peroxide. The chemical composition, specific surface area (SSA), and pore size of bagasse before and after pretreatment were investigated, while the experiments on the adsorption equilibrium of cellulose to cellulase and reagent reuse were also performed. Scanning electron microscopy (SEM) and high-performance liquid chromatography (HPLC) were employed for microscopic morphology observations and glucose analysis, respectively. The results showed that pretreated bagasse was deconstructed into cellulose with a nanofibril network, most of the hemicellulose (~100%) and lignin (~98%) were removed, and the SSA and void were enlarged 11- and 5-fold, respectively. This simple, mild preprocessing method enhanced cellulose accessibility and reduced the biological barrier of the noncellulose component to improve the subsequent enzymolysis with a high glucose recovery (98.60%).

## 1. Introduction

Lignocellulose biomass is an energy form that plants utilize to store energy gained via photosynthesis [1]. It is known as a carbon-neutral, green resource, with a total annual output of 146 billion tons [2]. Fuel, materials, and chemicals in solid, liquid, and gaseous states are obtained from biomass using various technical means [3,4,5]. However, biological barriers make it extremely difficult to release fermentable sugars from cellulosic biomass. As a result, large dosages of enzymes are required for hydrolysis, which diminish their cost-efficient features for commercial application [6]. Since the energy crisis in the 1970s, biofuel and biochemical production technologies based on enzymolysis from biomass have been driven in both industry and academia [7]. Although enzymolysis offers potentially higher yields [8], higher selectivity [9], lower energy costs [10], and milder operating conditions [11] compared to chemical processes, the technology still faces significant challenges. Eliminating the biological barriers of lignocellulose biomass to enzymes under mild conditions is vital [12].

Cellulose, as a linear polymer consisting of 300–15,000 D-glucose units, aggregates into 3 to 4 nm-wide elementary fibrils due to intermolecular forces [13,14,15]. The elementary fibrils are embedded in the hemicellulose matrix and are further aggregated into 10 to 25 nm-wide primary microfibrils [16]. These primary microfibrils are likewise embedded in a lignin–carbohydrate complex (LCC) matrix and are then bonded together, weaving throughout the plant cells in a unique way [17,18]. Limiting the accessibility of polysaccharides and unproductive binding to enzymes are the main mechanisms by which the unique icing that is formed by LCC limits the enzymolysis of lignocellulose biomass [19,20].

To reduce the cellulase required, the accessibility of cellulose through pretreatment by mechanical, chemical, biological, or a combination of these methods has been extensively studied [21,22]. Some early studies show that the dosage of catalyst needed was lower than 20 FPU/g. These pretreatment techniques can be divided into two types according to their mechanism: (1) Based on hydrolysis (dilute acid, hydrothermal, alkali, enzyme) and oxidation (basic hydrogen peroxide) mechanisms to cut off the molecular chain to remove noncellulose components by improving the mass transfer channels [23]. These methods are limited by the resistance of the plant cell wall; it is difficult to completely remove hemicellulose and lignin. (2) Based on mechanical forces (ball milling), molecular forces (ionic liquids, deep eutectic solvents (DESs), inorganic salt hydrates) destroy cellulose aggregation and increase cellulose exposure [24]. The main problem with this kind of method is that cellulose overdisperses or overdissolves and mixes with residual noncellulose components, which may show some deterioration over time [25]. Thus, assuming limited swelling of cellulose combined with hydrolysis and oxidation in one-pot treatment is expected to simultaneously achieve: the removing of the hemicellulose and lignin, and the increasing of cellulose accessibility, although this has not been reported.

H_3_PO_4_ is an effective cellulose dissolution and swelling agent that can be easily customized for cellulose intercrystalline swelling or dissolution, depending on the properties of cellulose and operating conditions. Walseth [26] first developed a high-reactivity cellulose for cellulase activity analysis by dissolving cellulose using H_3_PO_4_, which has become one of the most common cellulose substrates for cellulase activity analysis. Previous studies have shown that H_3_PO_4_ (with the help of H_2_O_2_) can extract nanofibrils and high-reactivity cellulose suitable for enzymolysis from biomass [27,28]. However, few studies have revealed the influence of intercrystalline swelling of cellulose on bagasse enzymolysis. 

In this work, bagasse was pretreated by using an H_3_PO_4_ and H_2_O_2_ aqueous solution system under mild conditions, which has three functions, namely, swelling, hydrolysis, and oxidation. Cellulose swelling, hemicellulose hydrolysis, and lignin oxidation degradation occur simultaneously during the pretreatment, and they cooperate and promote each other. Pretreated bagasse was deconstructed into cellulose with a nanofibril network, most of the hemicellulose (~100%) and lignin (~98%) were removed, their pore volume suitable for enzyme entry was magnified 11-fold, and their surface area available for cellulase loading was increased 5-fold. This preprocessing approach enhanced cellulose accessibility and reduced the barrier of noncellulose components to improve the subsequent enzymolysis with a high glucose recovery (98.60%). In addition, the used H_3_PO_4_ mixture can be reused for subsequent pretreatment or neutralized to produce a fertilizer rich in phosphorus [29]. This study demonstrates a strategy with simple, mild features, which has the potential pretreatment methods for bioethanol processing and a new possible pathway for biomass-refining technology development.

## 2. Experimental

### 2.1. Cellulose and Cellulase 

The bagasse used in this study was purchased from Guangxi Guitang Group Co., Ltd. (Guitang, China). The bagasse was ground to a 40–60 mesh powder. Analytically pure reagents, phosphoric acid (H_3_PO_4_, 85% *w*/*v*), anhydrous ethanol (98% *w*/*v*), and hydrogen peroxide (H_2_O_2_, 30% *w*/*v*) were purchased from Nanning Blue Sky Experimental Equipment Co., Ltd. (Nanning, China).

A total of 30 g bagasse powder, 60 mL H_2_O_2_, and 240 mL H_3_PO_4_ were placed in a round-bottomed flask and pretreated at 30 °C with stirring at 300 rpm for 42 h. After the reaction, the solids were recycled by filtration from the suspension and then soaked in 100 mL anhydrous ethanol for 24 h. The pretreated bagasse was recovered by centrifugation at 4000 rpm for 15 min from ethanol suspension.

The chemical composition of raw materials and pretreated bagasse was determined according to the standard method of the US National Renewable Energy Laboratory (NREL) [30]. This involved a two-stage extraction of samples followed by a two-stage acid hydrolysis. Residual solids were quantified as acid-insoluble lignin content. The acid-soluble lignin content was quantified by a UV spectrophotometer (Agilent Cary 3500, Agilent, Santa Clara, CA, USA) in the analytical hydrolysate. The lignin content was the sum of acid-soluble lignin and acid-insoluble lignin content. Structure of cellulose and hemicellulose were quantified as their monomeric forms in the analytical hydrolyzate using high-performance liquid chromatography (HPLC, Agilent 1260 Infinity Ⅱ, Agilent, Santa Clara, CA, USA) with an HPX-87H column (Agilent, Santa Clara, CA, USA). Cellulase (Novozyme CTec2) was purchased from Sigma-Aldrich (Shanghai, China). The cellulase activity was determined by the filter paper method according to US NREL [31] and protein content was determined using the Bradford method [32]. 

### 2.2. Physicochemical Properties of Cellulose

#### 2.2.1. X-ray Diffraction 

The X-ray diffraction (XRD) pattern was obtained using a MiniFlex 600 advance X-ray diffractometer (Rigaku, Tokyo, Japan) with a Cu Kα radiation source operated at 40 kV and 40 mA. The measurement of 2*θ* ranged from 10° to 50° at a scanning speed of 5°/min and step size of 0.02°. The crystallinity index (*CrI*) of pretreated cellulose was calculated by subtracting the amorphous contribution from diffraction spectra using an amorphous standard according to a previous study [33]. XRD was calculated by the following formula:

*CrI* (%) = (*I*_200_ − *I*_am_)/*I*_200_ (1)

where *I*_200_ represents the maximum intensity of the lattice diffraction peak at 2*θ* between 22.5°, and *I*_am_ represents the intensity scattered by the amorphous component in the sample, which was evaluated as the lowest intensity at 2*θ* at 18°.

#### 2.2.2. Degree of Polymerization 

The intrinsic viscosity degree of polymerization (DP) test [34] was used to calculate the DP of cellulose. The DP was calculated according to the following equation (with an average of three measurements per sample):

[*η*]*_G_* = *η*_sp_/*C* × (1 + 0.35 *η*_sp_) (2)

*DP* = 80 [*η*]*_G_* (3)

where [*η*]*_G_* is the intrinsic viscosity (mL/g), *η*_sp_ is the specific viscosity, *C* represents the concentration (g/100 mL), and *DP* is the degree of polymerization.

#### 2.2.3. Specific Surface Area 

Nitrogen adsorption (Micromeritics ASAP2460, Norcross, Georgia) was used to measure the specific surface area (SSA) of untreated and pretreated bagasse. The samples were degassed at 90 °C for 12 h prior to analysis to remove moisture and air from the substrate pores. The test was carried out at liquid nitrogen temperature, and the SSA of the sample was calculated using the BET model [35].

#### 2.2.4. Zeta Potential 

The surface charge of the pretreated cellulose was evaluated by determining the zeta potential using the zeta potential mode of the Malvern Zetasizer (ZS90X, Melvin, UK) [36]. The pretreated cellulose was uniformly dispersed in a sodium citrate buffer of pH 4.8 to form a 0.5% (*w/v*) suspension, and the suspension was measured and scanned with a cuvette 100 times. 

#### 2.2.5. Additional Measurements and Characterization 

An X-ray photoelectron spectrometer (XPS) (ULVAC-PHI, Chigasaki-shi, Japan) was used to determine the surface chemical analysis of pretreated cellulose [37]. A Fourier-transform infrared (FTIR) spectrometer (TENSOR II, Brook Technology, Ettlingen, Germany) was used to obtain the FTIR spectra of the untreated bagasse, pretreated bagasse, and enzymolysis residual in the frequency range of 4000–400 cm^−1^ with a resolution of 4 cm^−1^ using the KBr tablet method [38]. Scanning electron microscopy (SEM) SU8220 (Hitachi, Tokyo, Japan) was used to analyze the surface structure of the untreated bagasse and pretreated bagasse. ImageJ software (Version 2.0, National Institutes of Health, Bethesda, MD, USA) was used to determine the diameter of the nanofibers after at least 100 measurements based on SEM images. The bagasse samples were freeze-dried using the Advantage Plus EL-85 freeze-drying system (SP Scientific, Warminster, PA, USA) and the samples were sprayed with gold to improve the conductivity of the samples before observing the samples. 

### 2.3. Adsorption Equilibrium Experiment

Two hundred milligrams of substrate were weighed into a centrifuge tube and a series of concentrations of enzyme solutions were added (0.05 M citrate buffer, refrigerated at 4 °C before use) to form a solid loading of 2% (*w*/*v*). The mixture was shaken at 130 rpm at 4 °C for 2 h. In parallel, a blank control sample was run. After adsorption, the mixture was centrifuged at 10,000 rpm for 5 min, and the supernatant was taken. The protein concentration was determined by the Bradford method and each sample was measured in duplicate. The adsorption capacity was expressed as the difference between the concentration of added enzyme protein and that of supernatant. The adsorption data were fitted using the Langmuir equation [39]:

*E*_b_ = (*E*_bm_ × *K*_a_ × *E*_f_)/(1 + *K*_a_ × *E*_f_) (4)

where *E*_b_ is the amount of bound cellulase (mg/g substrate), *E*_bm_ represents the theoretical maximum adsorption capacity of the substrate (mg/g substrate), *K*_a_ is the affinity constant (L/mg), and *E*_f_ is the free enzyme in the supernatant (mg/mL).

### 2.4. Enzymatic Hydrolysis

Enzymolysis of pretreated bagasse was carried out in a 50 mM citrate buffer (pH 4.8) with a substrate load of 2% (*w*/*v*; dry matter, DM). Cellulase was introduced at 5, 10, and 20 filter paper unit (FPU)/g cellulose, and 0.1 g/L ampicillin trihydrate was added to avoid microbial interference during hydrolysis. After enzymolysis for 0.5, 2, 4, 8, 16, 48, and 72 h, ~5 mL of solid–liquid mixture was taken out and inactivated at 100 ℃ for 30 min, passed through a 0.22 μm filter membrane, and stored at 4 °C for further measurement of glucose yield. Enzymolysis of each sample (untreated and pretreated bagasse) was run in parallel. The glucose concentration was measured at 60 °C using an HPLC system equipped with an HPX-87H column (Agilent, Santa Clara, CA, USA). The mobile phase flow rate was at 0.6 mL/min and the detection time was 30 min. The hydrolysis efficiency of the enzyme bound to the cellulose surface was calculated by the hydrolysis rate of the unit bound enzyme in the initial stage of enzymolysis (0.5 h).

## 3. Results and Discussion

### 3.1. Physical and Chemical Property Characterization 

**Figure 1:**
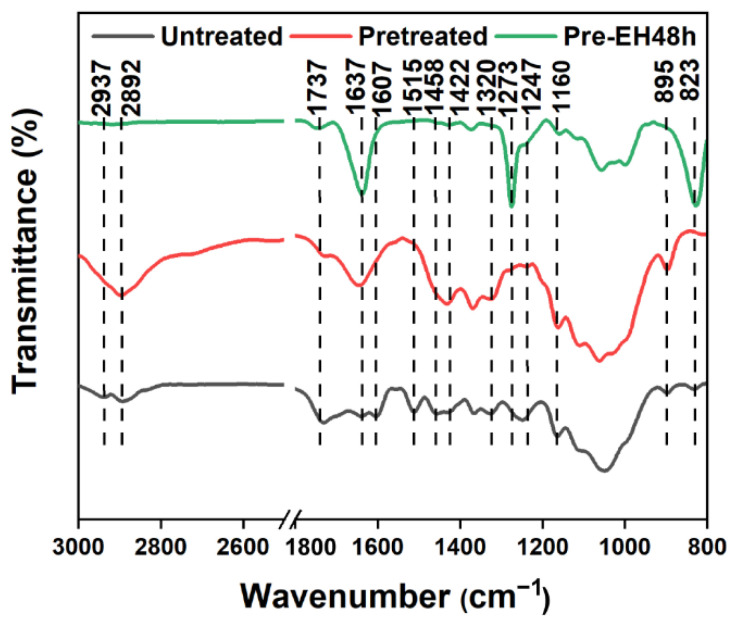
Fourier-transform infrared spectrometer (FTIR) spectrum of the untreated and pretreated bagasse.

To assess the efficacy of the pretreatment in the removal of noncellulose components, the chemical composition of untreated and pretreated bagasse is compared in Table 1. Table 1 showed that ~100% of initial hemicellulose in the bagasse was removed during the pretreatment. As shown in the FTIR results (Figure 1), the characteristic peaks at 1737 cm^−1^ (C=O stretching of the acetyl and urate groups of hemicellulose or the ester bond of carboxyl groups in lignin to fragrant acid and ferulic acid) and 1247 cm^−1^ (the alkyl ester of the acetyl group in hemicellulose) of the hemicellulose of pretreated bagasse from untreated bagasse are decreased or completely disappeared [40]. These indicate that hemicellulose removal is complete [41], which is attributed to the fact that cellulose intercrystalline swelling fully exposes the hemicellulose and promotes the hydrolysis of the hemicellulose. Similarly, 98% of the initial lignin was removed during the pretreatment (Table 1). As seen from the FTIR results (Figure 1), the characteristic peaks at 1515 cm^−1^ (C=C stretching of the aromatic skeleton), 1607 cm^−1^ (the aromatic skeletal stretching), and 1458 cm^−1^ (C–H deformation of CH_3_ and CH_2_) of lignin of pretreated bagasse from untreated bagasse almost disappeared [42]. This demonstrates that lignin was efficiently removed, attributing to the oxidative degradation of lignin by peroxyphosphoric acid (H_3_PO_5_) formed by H_3_PO_4_ and H_2_O_2_ [43]. The cellulose yield possibly reached 96.03% (Table 1), which is due to both the mild reaction conditions and high selectivity of the delignification and hemicellulose removal [43].

**Figure 2:**
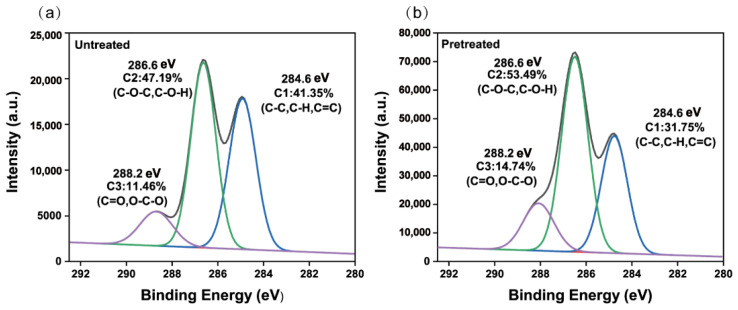
X-ray photoelectron spectrometer (XPS) spectrum of (**a**) untreated bagasse and (**b**) pretreated bagasse.

As shown in the XPS results (Figure 2a,b), the oxygen-to-carbon (O/C) ratio of untreated bagasse was 0.39. The known theoretical O/C ratios of cellulose, hemicellulose, and lignin are 0.83, 0.81, and 0.33, respectively. The low O/C ratio of natural bagasse can explain the lignin on the surface of the fibrils. The O/C ratio of pretreated bagasse increased to 0.62. The concentrations of C1 (C=C/C-C/C-H), C2 (C-O-C/C-O-H), and C3 (C=O/O-C-O) in untreated bagasse were 41.35%, 47.19%, and 11.46%, respectively. Contributions of cellulose, hemicellulose, and lignin to these peaks have been reported [44,45], with 85% of cellulose signaling to C2 and part of it to C3, 80% of hemicellulose signaling to C2 and the rest to C3, and 50% of lignin signaling to C1 and the rest to C2. The C1 content of pretreated bagasse decreased, while C2 and C3 contents increased. These phenomena suggest that the lignin is removed and the polysaccharides are exposed on the surface of the fibers [46]. This was consistent with the results of the chemical composition and FTIR analysis.

**Figure 3:**
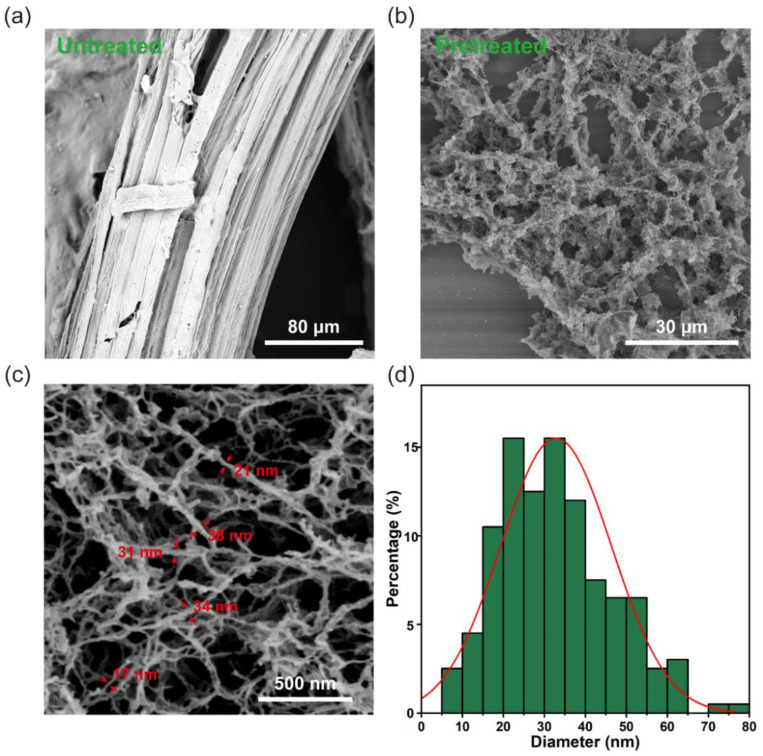
Scanning electron microscopy (SEM) images of (**a**) untreated bagasse and (**b**,**c**) pretreated bagasse; (**d**) diameter distribution of the nanofibers measured based on SEM images.

The structure of untreated bagasse is complete and compact, and the fiber bundles are arranged compactly (Figure 3a). This intact structure greatly impedes the accessibility of the cellulase to the cellulose. Bagasse was pretreated in an aqueous solution of H_3_PO_4_, and the surface morphology of the pretreated bagasse changed significantly, transforming the dense bagasse into cellulose with a nanofibrils skeleton network structure (Figure 3b,c). The widths of most nanofibers are in the range of 10–60 nm (Figure 3d). This is attributed to the fact that the H_3_PO_4_ molecules intrude between the fibrils, breaking the hydrogen bonds between adjacent fibrils [47]. The removal of hemicellulose and lignin also increases the number of channels for H_3_PO_4_ molecules to squeeze into the cell wall, causing the distance between adjacent fibrils to widen.

**Figure 4:**
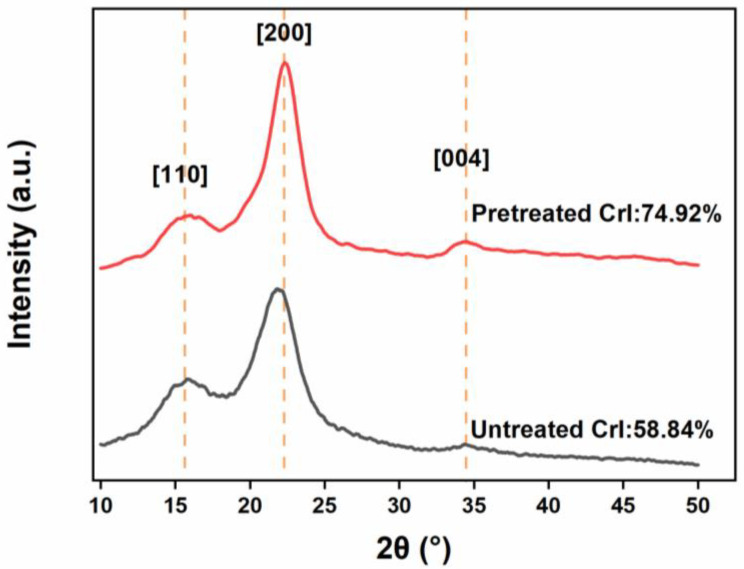
X-ray diffraction (XRD) patterns of the untreated bagasse and pretreated bagasse.

To evaluate the effect of pretreatment on cellulose aggregation, the XRD patterns of untreated and pretreated bagasse were compared (Figure 4). The peaks [48] at 16° (101), 22° (200), and 34° (004) for cellulose I were significantly strengthened in the XRD patterns of untreated and pretreated bagasse. Similarly, the CrI value of the pretreated bagasse increased from 58.84% to 74.92% (Figure 4). There were no obvious clear peaks at 2*θ* = 12.1° (110 for cellulose II), and 20.2° (110 for cellulose II) in the XRD patterns of pretreated bagasse as reported in the literature [49]. These indicate that the cellulose crystal structure was unchanged, and the supramolecular structure of cellulose was not visibly broken. This implies that the swelling of H_3_PO_4_ in cellulose mainly occurs in the intercrystalline spaces rather than the intracrystalline spaces.

### 3.2. Adaptability of Pretreated Bagasse to Cellulase

**Figure 5:**
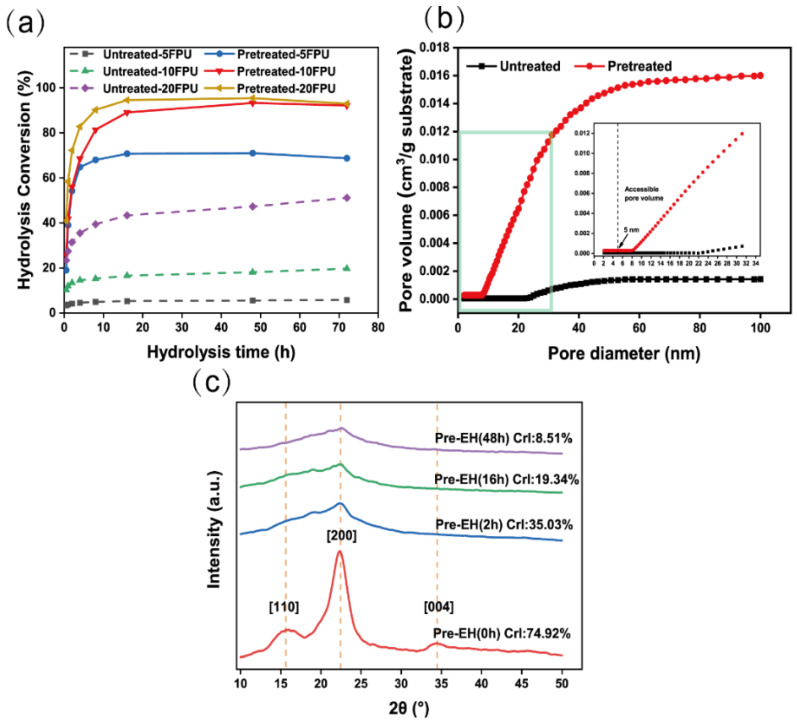
(**a**) Enzymolysis conversion of untreated and pretreated bagasse at 5, 10, and 20 FPU/g; (**b**) cumulative pore volume of untreated and pretreated bagasse; (**c**) X-ray diffraction (XRD) patterns of the pretreated bagasse enzymolysis for 0, 2, 16, and 48 h.

To evaluate the effect of cellulose intercrystalline swelling on bagasse enzymolysis, the adaptability of pretreated bagasse to cellulase was analyzed (Figure 5a). As seen from the enzymolysis of pretreated bagasse, the glucose yield (78.19%) achieved at a lower enzyme dosage of 5 FPU/g was 14-fold higher than that achieved with untreated bagasse (5.25%). Further increase in the cellulase dosage to 10 FPU/g resulted in a glucose yield of 95.91% that was five-times higher than that achieved with untreated bagasse (18.07%). However, with 20 FPU/g of cellulase, a glucose yield of 98.60% was obtained: this was two-fold higher than that achieved with untreated bagasse (47.27%). This indicates that pretreated bagasse is highly amenable to cellulase.

Highly selective removal of lignin (~98%) and hemicellulose (~100%) helps to reduce the unproductive adsorption and the physical barrier of bagasse to cellulase (Table 1). These, in addition to the lower noncellulose content of the pretreated bagasse, are also associated with changes in other physicochemical properties including [50,51] pore volume (PV), SSA, degree of polymerization (DP), and CrI, directly and indirectly providing information about enhanced enzymolysis of pretreated bagasse. As seen from the PV results (Figure 5b), a new mesopore (8–23 nm) appeared in the pretreated bagasse and the PV increased to 1.60 × 10^−2^ cm^3^/g from 1.43 × 10^−3^ cm^3^/g (Figure 5b). Pores larger than 5.1 nm allow the enzyme to enter the substrate without being restricted by size [52]. The PV of pretreated bagasse increased 11-fold, meaning that the physical channels through which the enzyme can pass are increased. The SSA of pretreated bagasse significantly increased to 1.9068 m^2^/g from 0.3633 m^2^/g (Table 2). The increase in the SSA of cellulose means that a larger area is available for enzyme loading [53]. The SSA of pretreated bagasse increased five-fold, meaning that the available surface area of the cellulose for enzyme loading was enhanced. The DP of cellulose dropped to 300 from an initial value of 2877 during the pretreatment (Table 2). This is attributed to the cleavage of the β-1,4 glycosidic bonds in cellulose by the acid-catalyzed hydrolysis during the pretreatment [54]. It can therefore be inferred that the cellulose was destroyed and depolymerized, meaning that the number of nodes requiring cellulase hydrolysis was reduced.

As shown in the XRD results (Figure 5c), the CrI value decreased sharply to 35.03% from 74.92% [55], while the corresponding cellulose conversion to glucose was 55.32% during the 2 h enzymolysis. When the enzymolysis time was extended to 16 h, the percentage of cellulose to glucose increased to 80.02% and the CrI value decreased to 19.34%. The remaining crystalline cellulose was greatly enzymolyzed, the glucose yield reached to 98.6%, and the CrI value of the residue dropped to 8.51%. The cellulase therefore showed a strong preference for the digestion of crystalline cellulose over amorphous cellulose. This may be because amorphous cellulose is mixed with noncellulose components, which hinders the approach of cellulase.

As shown in the XPS results (Table S1), the O/C ratio of the residual from the pretreated bagasse enzymolysis at 20 FPU/g for 48 h decreased from 0.62 to 0.31. In addition, the content of C1 increased (from 31.75% to 58.79%), and the content of C2 decreased (from 53.49% to 27.17%) (Figure S1). As seen from the FTIR results (Figure 1), the characteristic absorption peaks [56,57] at 895 cm^−1^ (the glycosidic bond of cellulose), 2892 cm^−1^ (C–H tensile vibration of methyl and methylene), 1160 cm^−1^ (C–O–C asymmetric stretching of cellulose), and 1066 cm^−1^ (C–O, C–C stretching vibration) of cellulose were weakened. The characteristic absorption peaks [58,59] at 823 cm^−1^ (C–H bending vibration of guaiacyl), 1273 cm^−1^ (C-O stretching vibration of guaiacyl), and 1637 cm^−1^ (C=O conjugated stretching) of lignin were significantly enhanced in the FTIR of the residue (Figure 1). This is attributed to the cellulose being converted to glucose (98.6%) by cellulase and being removed, while the lignin was retained in the enzymolysis residue. 

### 3.3. Enzymolysis Kinetic Behavior of Pretreated Bagasse

**Figure 6:**
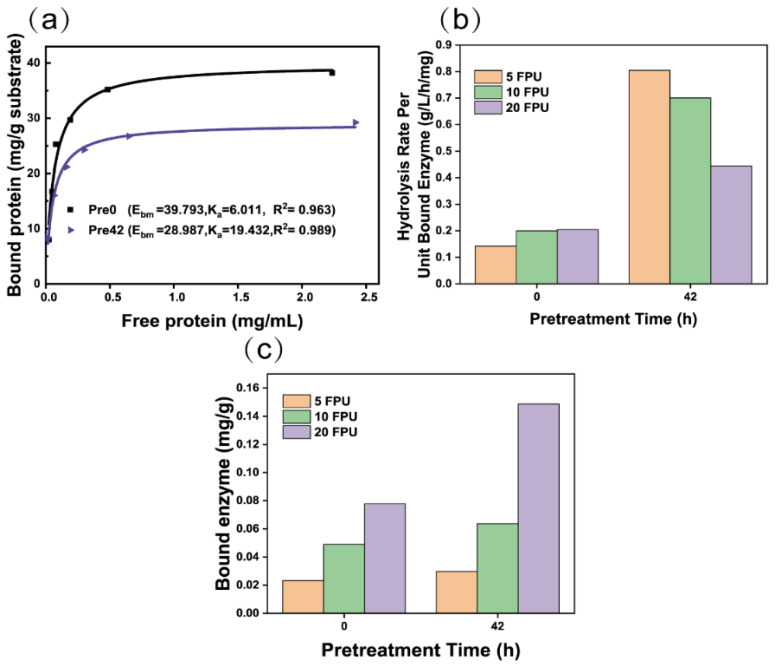
(**a**) Langmuir adsorption isotherms and parameters of cellulase onto pretreated bagasse at 4 °C, (**b**) initial hydrolysis efficiency, and (**c**) bound enzyme in pretreated bagasse with loading of 5, 10, and 20 FPU/g cellulase for 0.5 h.

The linear correlation coefficient (R^2^) was greater than 0.963, indicating that the Langmuir [39,60] equation fits the adsorption isotherm data well (Figure 6a). The affinity constant of pretreated bagasse was 19 L/g, which was three times that of untreated bagasse (6 L/g). This suggests that pretreated bagasse adsorption enzymes require a higher enzyme concentration at saturation than untreated bagasse. The adsorption capacity of cellulase onto the pretreated bagasse decreased to ~29 mg/g from ~40 mg/g (Figure 6a). The adsorption behavior of cellulase onto lignin is well-understood [61] and the hydrophobic lignin enhances the hydrophobic interaction, increasing the adsorption of enzymes onto lignin [62]. The hydrophobic interaction was weakened in pretreated bagasse due to the lower lignin content (Table 1). Cellulase was negatively charged in the buffer at pH 4.8 and demonstrated an electrostatic repulsion of cellulose, which had a negatively charged surface (−45.61 mV). Due to the weakening of the hydrophobic interaction and the enhancement of the electrostatic interaction of pretreated cellulose and cellulase, the adsorption capacity of cellulase onto cellulose decreased, although the SSA of the pretreated bagasse increased (Table 2).

The unit-bound enzyme efficiency was calculated based on the enzymolysis rate at 0.5 h (Figure 6b). The unit-bound enzyme efficiency of pretreated bagasse was significantly improved (Figure 4b), although the amount of bound enzyme onto bagasse remained unchanged (Figure 6c). This means that productive adsorption increases due to increased cellulose exposure to cellulase after the removal of noncellulose components (Table 1). This implies that the adequate removal of the noncellulose components is necessary for the enzyme to diffuse into or access the cellulose. The unit-bound enzyme efficiency (0.76 g/L/h/mg bound enzyme) of bagasse at low enzyme doses was significantly higher than that of the high enzyme doses of 10 and 20 FPU/g (0.68 and 0.43 g/L/h/mg bound enzyme). This suggests that pretreated bagasse is more conducive to enzyme efficiency at lower enzyme doses.

### 3.4. Evaluation of H_3_PO_4_ Recyclability

**Figure 7:**
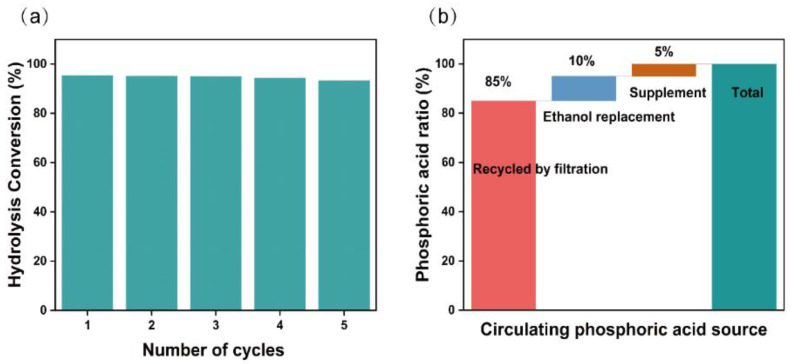
(**a**) Enzymatic hydrolysis conversion at 10 FPU/g for 48 h of bagasse pretreated with five cycles of H_3_PO_4_; (**b**) source of recycled H_3_PO_4_.

‘Green’ and sustainable production is widely recognized by human society [63]. If the final production quality is not disturbed, the reuse of reagents for pretreatment can significantly reduce the cost. The H_3_PO_4_ mixture was reused five times for subsequent pretreatment of bagasse with the appropriate addition of H_3_PO_4_, and the resulting glucose yield of pretreated bagasse via enzymolysis was similar to that of the fresh reagent (Figure 7a). Approximately 85% of the H_3_PO_4_ is recycled directly by filtration, ~10% H_3_PO_4_ is recovered from ethanol washing solution via rotary evaporation, and only ~5% H_3_PO_4_ needs to be replenished for reuse (Figure 7b). Residual H_3_PO_4_ (~5%) in pretreated bagasse almost obviated the need for acid to adjust the pH to 4.8 to meet the requirements of the enzymatic hydrolysis process. Further research will be conducted to recover acid-soluble lignin from the H_3_PO_4_ mixture to further improve the reuse potential of H_3_PO_4_. 

In addition, phosphorus, in the form of phosphate, is an important nutrient for living things [64]. The treated filtrate rich in H_3_PO_4_ has the potential to be converted to phosphorus-rich fertilizer by reacting it with calcium hydroxide or ammonia water [65]. These examples indicate biomass pretreatment can be affected with milder environmental consequences.

## 4. Conclusions

Obtaining green energy and materials from renewable biomass is an indispensable pathway for human society to deal with the energy crisis and environmental issues. To overcome the biological barriers to biomass, a strategy of intergranular swelling of cellulose combined with hydrolysis and oxidation was proposed and demonstrated, which was used for enhancing the release of fermentable sugars by enzymolysis. Due to the fact that cellulose swelling, hemicellulose hydrolysis, and lignin oxidation degradation occur simultaneously during the pretreatment, they cooperate and promote each other. Bagasse was converted into cellulose with a nanoscale size, low DP, high void fraction, and high SSA. The cellulose in pretreated bagasse was sufficiently exposed to cellulase, affording a high glucose yield (98.60%), posing a competitive pretreatment method for enzymatic hydrolysis of biomass. This nanofiber network structure of cellulose provides the possibility for the combined production of fermentable sugars and nanocellulose, which will greatly improve the efficiency of biomass refining and inspire the development of novel cellulose-based materials. In addition, this study found that cellulase preferred crystalline cellulose to amorphous cellulose, which provided new evidence for further understanding the enzymatic hydrolysis mechanism of cellulose.

## Figures and Tables

**Table 1:** Composition of bagasse and pretreated bagasse.

	Yield (%) ^a^	Cellulose (%)	Hemicellulose (%)	Lingin (%)
Untreated bagasse	100/100	43.26 ± 2.13	22.86 ± 0.97	25.53 ± 1.18
Pretreated bagasse	42.58/96.03	97.56 ± 4.38	0	1.35 ± 0.11

^a^ Yield based on the initial amount of biomass/yield based on the initial amount of cellulose in biomass.

**Table 2:** Physicochemical properties of bagasse and pretreated bagasse.

	SSA (m^2^/g)	DPn	Zeta Potential (mV)
Untreated bagasse	0.3633 ± 0.016	2876.95 ± 26.83	−9.01 ± 0.59
Pretreated bagasse	1.9068 ± 0.207	300.6 ± 7.48	−45.61 ± 1.13

## Data Availability

The data presented in this study are available on request from the corresponding author.

## Supplementary Materials

The following supporting information can be downloaded at: https://www.mdpi.com/article/10.3390/polym14173587/s1, Figure S1: X-ray photoelectron spectrometer (XPS) spectrum of residue from enzymolyzed pretreated bagasse for 48 h, Table S1: oxygen/carbon (O/C) ratio of the X-ray photoelectron spectrometer (XPS) spectrum.
