# Supplementary material for: Enhancing for Bagasse Enzymolysis via Intercrystalline Swelling of Cellulose Combined with Hydrolysis and Oxidation
Source: Polymers (Basel). 2022 Aug 30;14(17):3587. doi: 10.3390/polym14173587 (PMC9460872; doi:10.3390/polym14173587)
Supplement: Supplementary file 1 [file polymers-14-03587-s001.zip › polymers-1878163-supplementary.pdf]

Supplementary Material

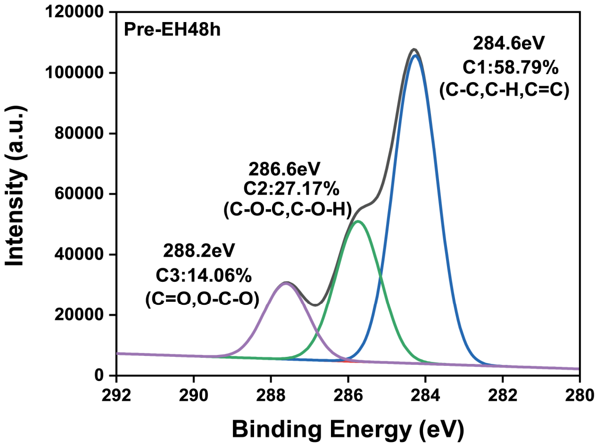

Figure S1. X-ray photoelectron spectrometer (XPS) spectrum of residue from enzymolyzed pretreated bagasse for 48 h.

Table S1. Oxygen/carbon (O/C) ratio of the X-ray photoelectron spectrometer (XPS) spectrum.

Substrates	O/C	C1 (%)	C2 (%)	C3 (%)
Untreated	0.39	41.35	47.19	11.46
Pretreated	0.62	31.75	53.49	14.74
Pretreated-EH (48 h)	0.31	58.79	27.17	14.06
